# Quality of life assessment instruments in children and adolescents with neuromuscular diseases: a systematic scoping review

**DOI:** 10.1186/s12955-024-02232-3

**Published:** 2024-02-16

**Authors:** Karoliny Lisandra Teixeira Cruz, Isadora Cristina Sousa Santos, Cyntia Rogean de Jesus Alves de Baptista, Ana Claudia Mattiello-Sverzut

**Affiliations:** Department of Health Science, Ribeirão Preto Medical School, University of São Paulo, Av. Miguel Covian, 120, Ribeirão Preto, São Paulo, 14.049-900 Brazil

**Keywords:** HRQOL, Health-related quality of life, Neuromuscular disease, Children

## Abstract

**Objective:**

(1) To identify instruments used to assess quality of life (QoL) in children and adolescents with neuromuscular diseases; (2) To identify the psychometric properties contained in these instruments.

**Methods:**

This is a scoping review in which the electronic databases Embase, Scielo, Scopus, Pubmed and Lilacs were used as well as grey literature. The following terms were used in the search for articles published in the last 10 years: children, adolescents, neuromuscular disease, and quality of life.

**Results:**

In total, 15 articles were included and evaluated, indicating 7 instruments used to assess QoL (PedsQL™ Inventory 3.0 Neuromuscular Module, the PedsQL™ 4.0, the PedsQL DMD Module, the PedsQL ™ MFS, the SOLE, the KIDSCREEN and the LSI-A). The number of items ranged from 17 to 45. In addition, 6 instruments showed psychometric properties, but only 2 showed good and high quality, either in internal reliability or reproducibility.

**Conclusion:**

Our results were able to map the main QoL assessment instruments of children and adolescents with neuromuscular disease and the most cited instrument was the PedsQL™ Inventory 3.0 Neuromuscular Module. Larger studies that assess psychometric properties and that are validated for most diseases are needed.

**Supplementary Information:**

The online version contains supplementary material available at 10.1186/s12955-024-02232-3.

## Introduction

Quality of life (QOL) is defined as “an individual's perception of their position in life in the context of the culture in which they live and in relation to their goals, expectations, standards and concerns” (World Health Organization (WHO) [1]. Some of the elements that can determine QoL are unrelated to health, such as the context of the individual and social relationships (spirituality, family, employment, and friends). Additionally, health-related elements (functional, mental, physical, and emotional well-being) should be highlighted [2]. The WHO concept of QoL is well accepted. However, there is a debate about QoL and health-related quality of life (HRQoL). Thus, HRQoL elucidates the self-perception of well-being aspects that may be related to diseases, symptoms, and treatment [3].

Despite this ongoing conceptual discussion, QoL can vary according to individual experiences [4, 5] with different instruments used to assess relevant information, such as diseases and disabilities and treatment effectiveness [3].

In children and adolescents with disabilities, QoL assessment has been widely used to identify facilitators and barriers and to select individualized therapeutic approaches [6–8].

However, QoL assessments need to include aspects of holistic well-being and not be limited to health aspects. QoL and the International Classification of Functioning, Disability, and Health (ICF) are connected since their base is a dynamic model that informs different aspects of the life of an individual with a disability [8, 9].

For children and adolescents with disabilities, incorporating the concepts of F-words (function, family, fitness, fun, friends, and future) in the context of the ICF reinforces the need to consider participation [10]. In this way, it is clear that the concept of QoL is contained in the F-words, and completes the health professionals’ approach [8, 10].

In neuromuscular diseases (NMDs), QoL remains compromised despite technological advances and improvements in drug treatments [11, 12]. NMDs are a set of conditions that can be hereditary and progressive with the involvement of one or more components, such as anterior horn cells, motor units, motor neurons, peripheral nerve, neuromuscular junction, and/or muscle [13–15]. In general, NMDs present muscle weakness, fatigue, respiratory problems, and scoliosis, which generate disabilities and directly affect daily activities, participation, and QoL [13, 14, 16]. The most common pediatric NMDs include Charcot-Marie-Tooth disease (incidence of one in 2,500 individuals), Duchenne muscular dystrophy (incidence of one in 3,500 boys), and spinal muscular atrophy (incidence of one in 10,000 individuals) [17, 18].

It is essential to investigate QoL given the severity of neuromuscular diseases and the impacts caused to the patient and the family, depending on the environment and culture [19]. In this context, QoL can be an indicator of the therapies’ effects and it can be used to make professional decisions [20].

Recently, several studies have mentioned QoL as an outcome in rehabilitation assessment and intervention [2, 16, 21–25]. However, there is limited knowledge regarding specific QoL assessment tools for the pediatric population, children, and adolescents with NMDs. Therefore, this study has mapped QoL instruments from the last 10 years that have been used in pediatric neuromuscular diseases. Secondarily, this study has explored information about the psychometric properties of the instruments.

## Methodology

### Structure of the study

To ensure the originality of this scoping review, the major databases have been consulted (PubMed, Cochrane Database of Systematic Reviews, and PROSPERO). This study was registered with Open Science Framework (10.17605/OSF.IO/6J9PZ.).

### Search strategies and databases

This study was conducted according to methodology proposed by the Joanna Briggs Institute Reviewer's Manual [26]. The guiding question was defined as, "What are the most widely used instruments in the world to assess the quality of life of children and adolescents with neuromuscular diseases? Which of these instruments are validated by the pediatric population?".

To answer this question, the acronym PCC was used to scope review: Participants (children and adolescents with neuromuscular disease), the Concepts (quality of life), and the Context (health).

For the extraction of descriptors, the vocabularies were consulted in the search tools DeCs (Descriptors in Health Sciences) and MeSH (Medical Subject Headings). The descriptors used were: Children, child, infant, adolescents, teenager, neuromuscular disease, neuromuscular disorder, neuromuscular and quality of life and the Boolean terms AND and OR were used to compose the search keys (Supplementary material 1).

The searches took place in July, August and September 2022, and were conducted in 6 electronic databases: Embase, Scielo, Scopus, Pubmed, and Lilacs. Gray literature was searched on Google and Google Scholar, using the same descriptors and pdf type files.

### Eligibility

The eligibility assessment involves both the screening and the confirmation of the articles obtained in the searches [26]. For this, it is necessary to choose some criteria, which were determined according to the characteristics of the acronym PCC. Articles should focus on children and adolescents with NMDs and tools that address patient and caregiver perspectives on QoL.

Therefore, experimental, descriptive and observational articles, published in English, Portuguese and Spanish in the last ten years (2012 to 2022), addressing the quality of life of children and adolescents (age 0 to 18 years) with clinical diagnosis of neuromuscular disease were included.

The excluded studies were those focused on different age groups (adults and children), those that investigated the QoL of the caregiver and/or family but not the patient, those that provided limited information regarding the QoL instrument, those related to cancer diagnosis, as well as unpublished dissertations, clinical trials of medications/therapies, and surgeries (pre- and post-operative).

### Data screening and extraction

The screening process consists of surveying, organizing and compiling all the results obtained from the databases. For this, the Mendeley Reference Manager tool was used to merge all the search results and remove duplicates.

Data screening was conducted by two reviewers (KLTC and ICSS) independently, and they first read the titles and abstracts of the articles. Subsequently, they selected articles for reading considering the eligibility criteria listed above. Then, an excel spreadsheet was created containing the main data of each study: (I) Characteristics of the study (title, author, year of publication, language, country of origin, type of study, objective); (II) Characteristic of the sample age, neuromuscular clinical diagnosis, sample size; (III) Characteristic of the QoL instrument (instrument, components, form of application, score/stratification and quality of life outcome). Disagreements and doubts were resolved by a third reviewer (CRJAB).

## Results

### Characteristics of the studies

**Fig. 1:**
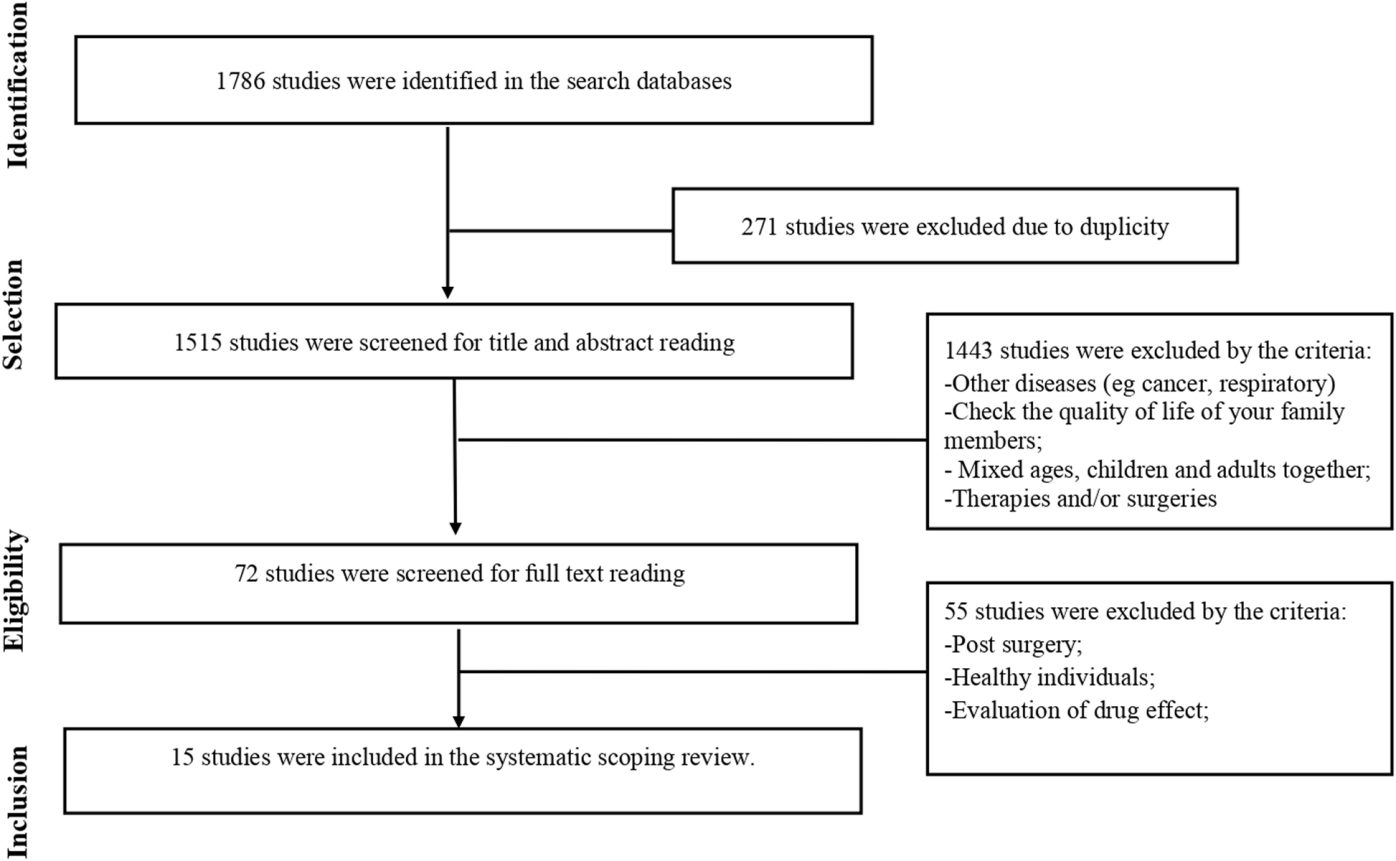
Flowchart of information from the phases of the systematized scope-type review

One thousand seven hundred and eighty-six (1786) articles were identified in the databases and 72 articles were selected, following the eligibility criteria. Among the 72 articles that had a full reading, 15 were included in this review (publications between 2012 to 2022, according to Fig. 1). The types of studies found were: 1 cross-sectional observational [27], 1 cohort [28], 1 prospective longitudinal multicenter cohort [29], 2 cross-sectional [30, 31], 1 cross-sectional descriptive [32], 1 observational, analytic and prospective [33], and 8 unreported [34–41].

### Country of origin of the studies

The studies were conducted in several countries, including 3 articles in the USA [28, 34, 40], 2 in China [35, 41], 2 in Italy [29, 38], 2 in Thailand [30, 31], 1 in Argentina [33], 1 in Brazil [40], 1 in Chile [27], 1 in Iran [39], 1 in the Czech Republic [36], and 1 in Turkey [32].

### Participants and sample

The number of participants assessed in the studies ranged from 35 [36] to 221 [39], and the participants were between 2 and 18 years old.

Of the scientific articles that composed this review, 8 studies were developed exclusively with patients who had Duchenne muscular dystrophy (DMD) [28, 29, 31, 34, 35, 39, 41]; 2 studies were developed with patients who had spinal muscular atrophy (SMA) types I, II and III [27, 36]; one study consisting of patients with DMD and Becker muscular dystrophy [32]; one study with patients with DMD, girdle muscular dystrophy and spinal muscular atrophy [40]; one study with patients with DMD, spinal muscular atrophy, Charcot-Marie-Tooth disease, and other neuromuscular diseases [30] and, finally, 2 studies consisting of a collection of NMDs [33, 38] (Table 1).

**Table 1:** Quality of life instruments (*n* = 15)

Authors/ Year	Title	Country	Study design	Sample size/ Age	Disease	QoL Instruments
Vega, et al., 2020 [27]	Quality of life in children and adolescents with Spinal Muscular Atrophy (Calidad de vida en niños y adolescentes con Atrofia Muscular Espinal)	Chile	Cross-sectional observational	*n* = 38 2 to 18 years	Spinal Amiotrophy I, II, III	Pediatric Quality of Life (PedsQLTM) Inventory 3.0 Neuromuscular Module
Bendixen, et al., 2012 [28]	Participation and quality of life in children with Duchenne muscular dystrophy using the International Classification of Functioning, Disability, and Health	USA	Cohort	*n* = 50 5 to 15 years *n* = 25 5 to 15 years	Duchenne muscular dystrophy Healthy children	Pediatric Quality of Life Inventory TM 4.0 (PedsQL)
Messina, et al., 2016 [29]	Health-related quality of life and functional changes in DMD: A 12-month longitudinal cohort study	Italy	Prospective longitudinal multicenter cohort	*n* = 98 5 to 13 years	Duchenne muscular dystrophy	Pediatric Quality of Life Generic Core Scale (PedsQL TM 4.0) Pediatric Quality of Life Inventory 3.0 Neuromuscular Module (PedsQL TM)
Thongsing, et al., 2020 [30]	Reliability and validity of the Thai pediatric quality of life inventory™ 3.0 neuromuscular module	Thailand	Cross-sectional	*n* = 103 2 to 18 years	Duchenne muscular dystrophy Charcot-Marie-Tooth Spinal Amyotrophy Other neuromuscular diseases	Pediatric Quality of Life Multidimensional Fatigue Scale (PedsQL TM MFS)
Thongsing, et al., 2019 [31]	Reliability and validity of the Thai version of the Pediatric Quality of Life inventory™ 3.0 Duchenne Muscular Dystrophy module in Thai children with Duchenne Muscular Dystrophy	Thailand	Cross-sectional	*n* = 56 5 to 18 years	Duchenne muscular dystrophy	Pediatric Quality of Life Inventory 3.0 Neuromuscular Module (PedsQLTM)
Köken, et al., 2021 [32]	Clinical features and quality of life in duchenne and becker muscular dystrophy patients from a tertiary center in Turkey	Turkey	Cross-sectional descriptive	*n* = 20 8 to 18 years *n* = 20 8 to 18 years	Duchenne muscular dystrophy Becker muscular dystrophy Healthy children	Módulo DMD do PedsQL™ 3.0 Pediatric Quality of Life Inventory (PedsQL) 4.0
Mozzoni, et al., 2021 [33]	Pediatric Quality of Life Inventory™, Neuromuscular Module, version 3.0 in Spanish for Argentina	Argentina	Observational, analytical and prospective	*n* = 185 2 to 18 years	Duchenne muscular dystrophy Other types of muscular dystrophy Spinal Amyotrophy Other myopathies	Pediatric Quality of Life (PedsQLTM) Inventory 3.0 Neuromuscular Module
Uzark, et al., 2012 [34]	Health-Related Quality of Life in Children and Adolescents With Duchenne Muscular Dystrophy	EUA	-	*n* = 117 6 to 18 years	Duchenne muscular dystrophy	PedsQL TM 4.0 Generic Core Scales (GCS)
Hu, et al., 2013 [35]	Reliability and validity of the Chinese version of the pediatric quality of life inventoryTM (PedsQLTM) 3.0 neuromuscular module in children with Duchenne muscular dystrophy	China	-	*n* = 56 2 to 18 years	Duchenne muscular dystrophy	Pediatric Quality of Life (PedsQLTM) Inventory 3.0 Neuromuscular Module
Kocova, et al., 2014 [36]	Health-related quality of life in children and adolescents with spinal muscular atrophy in the Czech Republic	Czech Republic	-	*n* = 35 3 to 18 years	Spinal Amyotrophy I, II, III	Pediatric Quality of Life (PedsQLTM) Inventory 3.0 Neuromuscular Module
Lim, et al., 2014 [37]	The level of agreement between child self-reports and parent proxy-reports of health-related quality of life in boys with Duchenne muscular dystrophy	USA	-	*n* = 63 6 to 16 years	Duchenne muscular dystrophy	Pediatric Quality of Life Inventory (PedsQL) 4.0
Orcesi et al., 2014 [38]	A New Self-Report Quality of Life Questionnaire for Children with Neuromuscular Disorders: Presentation of the Instrument, Rationale for Its Development, and Some Preliminary Results	Italy	-	*n* = 78 5 to 13 years *n* = 81 5 to 13 years	Genetic disorders Nervous disorders Spinal Amyotrophy Healthy children	SOLE Questionnaire
Zamani, et al., 2016 [39]	The quality of life in boys with Duchenne muscular dystrophy	Iran	-	*n* = 85 8 to 18 years *n* = 136 8 to 18 years	Duchenne muscular dystrophy Healthy children	KIDSCREEN
Simon, et al., 2017 [40]	Translation and validation of the life satisfaction index for adolescents scale with neuromuscular disorders: LSI-A Brazil	Brazil	-	*n* = 82 5 to 18 years	Duchenne muscular dystrophy Waist muscular dystrophies Spinal muscular atrophy	Life Satisfaction Index for Adolescents scale( LSI-A)
Liang, et al., 2019 [41]	Health-related quality of life in Chinese boys with Duchenne muscular dystrophy and their families	China	-	*n* = 15 2 to 18 years *n* = 15 2 to 18 years	Duchenne muscular dystrophy Healthy children	Pediatric Quality of Life Inventory (PedsQL) 4.0 (Chinese)

### Quality of life instruments

**Fig. 2:**
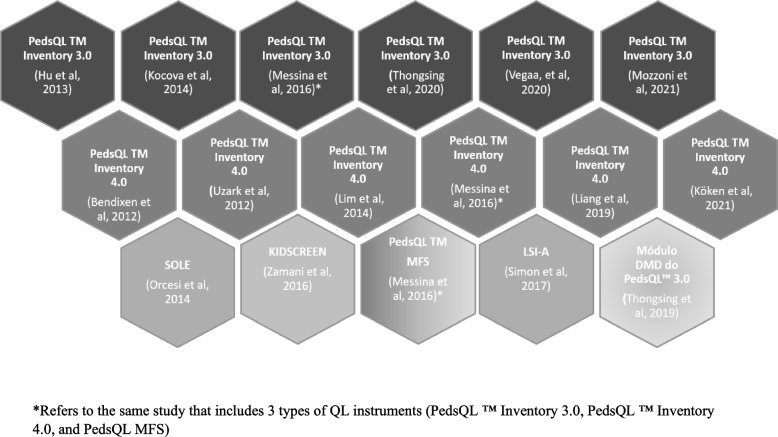
Mapping results on quality-of-life instruments in children and adolescents with neuromuscular disease

In this review 7 different instruments that assessed the quality of life of children and adolescents with neuromuscular diseases were found. The "Pediatric Quality of Life Inventory 3.0 Neuromuscular Module (PedsQL™ 3.0)" was used in six (*n* = 6) studies [27, 29, 30, 33, 35, 36] and the "Pediatric Quality of Life Inventory TM 4.0 (PedsQL™ 4.0)" was also used in other six (*n* = 6) [28, 29, 32, 34, 37, 41] studies. The first instrument consists of 25 items and the second instrument consists of 23 items. The other instruments were used in single studies: the DMD Module of the Pediatric Quality of Life (PedsQL™) [31], PedsQL™ MFS [29], SOLE questionnaire [38], KIDSCREEN [39], and Life Satisfaction Index for Adolescents (LSI-A) scale [40] (Fig. 2).

### Instrument domains

According to Table 2, the number of items contained in each instrument ranged from 17 to 45. The common domains among the instruments [28–30, 34, 40, 41] are physical, social, and psychological. The PedsQL 3.0 [27, 29, 30, 33, 35, 36] looks at disease, communication and family resources. The PedsQL™ Inventory 4.0 Neuromuscular Module and the PedsQL 4.0 Generic Core Scales [28, 29, 34, 37, 41] also include domain on school aspects. The PedsQL DMD Module [31] investigates barriers to treatment. And the PedsQL™ MFS [29] checks fatigue symptoms, with domains regarding sleep and rest fatigue, general fatigue, and cognitive fatigue. The KIDSCREEN [39] looks at domains of autonomy and relationship with parents, as well as social support. The LSI-A, finally, explores the child's personal satisfaction, leisure, and recreation [40].

**Table 2:** Characteristics of instrument reliability and validity

QoL Instruments	Number of studies	Number of items	Outcome Domains	Application mode	Scale construction	Language	Reability	Validity
PedsQL TM Inventory 3.0 Neuromuscular Module [27, 29, 30, 33, 35, 36]	6	17 (2 to 4 years) 25 (8 to 18 years)	Disease Communication Family resources	Patient and responsible interview	3 points—Likert scale (2 to 4 years questionnaire) 5 points- Likert scale (8 to 18 years questionnaire)	Spanish [27, 33] Italian [29] Thai [30] Chinese [35] Czech [36]	Internal consistency reliability(α = 0.7) [30] Stability reliability/ test–retest (ICC 0.69–0.82) [30] Internal consistency reliability(α = 0.7) (ICC 0.82–0.87) [33] Stability reliability/ test–retest (α = 0.7) (ICC 0.67–0.87) [33]	Construction validity [30] Construction validity [33]
PedsQL ™ Inventory 4.0 Neuromuscular Module [28, 29, 32, 34, 37, 41]	6	23	Physical Emotional Social School	Patient and responsible interview	3 points—Likert scale (5 to 7 years questionnaire) 5 points- Likert scale ( 2 to 4 and 8 to 18 years questionnaire)	Italian [29] Turkish [32] English [28, 37, 42] Chinese [41]	Internal reliability (α ≥ 0.7) [34] Stability reliability (ICC 0.74–0.88) [34]	-
PedsQL™ Módulo DMD 3.0 [31]	1	18	Activities of daily living Treatment barriers Preoccupation Communication	Patient and responsible interview	5 points/ Likert scale	Thai	Stability reliability/ test–retest (ICC 0.74–0.88)	Content validity
SOLE [38]	1	33	Self-perception of race Health status Level and quality of physical activity Overall performance Social and family participation Feelings	Interview patient and parents/ caregivers	3 points/ Likert scale	Italian	-	-
KIDSCREEN [39]	1	27	Physical activity and health General mood and feelings Famíly and free time Friends School and learning	Completion of the questionnaire (self-administered) by the patient and guardian	5 points/ Likert scale	Persian	-	-
LSI-A [40]	1	45	General welfare Interpersonal relationship Development Personal satisfaction Leisure and recreation	Patient Interview	5 points/ Likert scale	Portuguese (Brazil)	Reproductibility (ICC = 0.69–0.80) Reliability internal consistency (α = , 0.87 e 0.89)	-

### Instrument reliability and validity

Only 6 studies [30, 31, 33–35, 40] provided figures on reliability and validity. Three studies used the PedsQL™ Inventory 3.0 Neuromuscular Module questionnaire [30, 33, 35] and showed reasonable internal reliability (Cronbach's alpha ≥ 0.7) [43], stability reliability (test and retest) considered satisfactory (ICC 0.69–0.82 [30]; ICC 0.66–0.88 [35]; ICC 0.67–0.87 [33]) [44] and content validity [30, 33]. The study that used the Pediatric Quality of Life Generic Core Scale (PedsQL™ 4.0) [34] also described reasonable internal reliability (Cronbach's alpha ≥ 0.7), satisfactory stability reliability (ICC 0.74–0.88) with content validity. The study that used the LSI-A instrument [40] showed good internal reliability (Cronback's alpha ≥ 0.87) and satisfactory reproducibility (ICC = 0.69–0.80). Finally, the study that used the PedsQL™ DMD Module instrument [31] obtained satisfactory reproducibility (ICC 0.74–0.88) and showed content validity (Table 2).

## Discussion

This study revealed 7 instruments used to assess QoL in children and adolescents with NMD that were similar in some domains [28, 29, 31, 38, 39, 45, 46], two of which showed good reliability and validity. The neuromuscular disease with the greatest coverage in terms of QoL scales was DMD. The instrument with the highest frequency of use and applicable in the multiple pathological conditions was PedsQl [27, 29, 31, 32, 37, 42].

The different QoL assessment tools were applied to children and adolescents with neuromuscular diseases of various etiologies such as SMA [27, 30, 33, 36, 38, 46], DMD [28–33, 35, 37, 39, 41, 42, 46] and girdle dystrophies [46] and Charcot-Marie-Tooth disease [30]. Heterogeneous clinical presentations and age are factors to be considered in the choice of the QoL assessment tools. The instruments are available for children from two to six years old, who have demonstrated the ability to understand and self-report information about different aspects of their lives, independently [47, 48]. Similarly, children with asthma were aware of their disease and disabilities and could infer aspects of psychological and social nature [49]. Hospitalized children were also investigated and exhibited the ability to evaluate issues related to their lives [50]. However, almost all agreement between the self-reports of caregivers and the children is poor, with the latter having a more positive view about the QoL [38, 51–53]. These data reinforce the relevance of analyzing both the children’s and the caregivers’ perspectives.

Regarding psychometric properties, it is worth highlighting that PedsQL™ 4.0 was cross-culturally adapted in several countries [53–58]. Its Brazilian version applied to children with rheumatic diseases and healthy children [53] showed good reliability and validity, similarly to the original American version [55, 59, 60]. The correlation analysis between patients' and parents' answers ranged from moderate to weak (*r* = 0.77 for the physical dimension; *r* = 0.73 for the school dimension, and *r* = 0.49 for the emotional dimension and *r* = 0.59 for the social dimension) [53].

Agreement between parents and children also seems to be low in other versions of PEDSQL [52] and in other tools [38, 51]. A French study using PEDSQL 3.0 found that caregivers scored worse QoL than the DMD children [52]. In contrast, the PedsQL™ MFS applied to Turkish children with DMD and their parents showed moderate to strong agreement (ICC 0.84–0.91). It is worth highlighting that the PedsQL™ MFS version was reliable for assessing a relevant clinical symptom: the perception of fatigue [61].

Finally, the SOLE and LSI-A are instruments aimed at assessing QoL in the various NMDs. The SOLE proved to be a valid and reliable instrument, with good agreement (Pearson's correlation coefficient 0.365; *p* < 0.001) when compared to PEDSQL results for children born premature and typical [62]. The LSI-A has been shown to be sensitive to perceived changes in QoL [46, 63]. In children with DMD, the LSI-A instrument proved to be efficient in the qualification and quantification for its coverage of the various domains [40] including identifying limitations in physical condition, activities and social participation [64].

Based on the information obtained in this review, there are specific instruments to evaluate the QoL of the pediatric population with neuromuscular diseases. There is no tool that covers all domains of interest for a certain neuromuscular disease, i.e. fatigue is an item that appears in the PedsQL™ MFS [29] and children's personal satisfaction, leisure, and recreation only appear in the LSI-A [46]. Therefore, it is not possible to recommend one tool over another. According to our understanding, the clinician or researcher should select the assessment tool that presents robust psychometric properties and, if necessary, combine information of tools whose domains complement each other.

### Strengths and limitations of the study

This study has strengths: (a) it identified quality-of-life assessment tools focused on children and adolescents with neuromuscular diseases strictly outpatient; (b) the search process considered the main and most accessed electronic databases, including the gray literature, enabling a greater volume of articles consulted; (c) available data about the psychometric proprieties were described.

As limitations, the following can be listed: (1) studies in languages other than English, Spanish, and Portuguese were not included; (2) from the set of selected articles, only 6 articles provided data on the psychometric properties and may not reflect the best instruments for understanding the QoL of children and adolescents with neuromuscular disease.

### Recommendations

Considering the current view of QoL, a holistic indicator of the individual, the diversity of clinical presentations of pediatric NMD and the limited number of studies that evaluated QoL in this population, it is relevant that more scientific studies address this issue and focus on the analysis of the instruments’ psychometric properties.

## Conclusion

The results of this review suggest that the PedsQL™ DMD Module and LSI-A stood out as being the most suitable instruments to assess the QoL of children and adolescents with DMD, girdle dystrophies, and SMA, taking into account their psychometric properties. However, such instruments have not been tested in other myopathies and neuropathies.

The main contribution of this review is to gather the available instruments capable of investigating QoL in children/adolescents with neuromuscular diseases that are easy to apply and low cost, and sensitive to patients and caregivers/parents.

### Supplementary Information

**Additional file 1.**

## Data Availability

The datasets supporting the conclusions of this study is available from the corresponding author upon reasonable request.
