# Supplementary material for: Quality of life assessment instruments in children and adolescents with neuromuscular diseases: a systematic scoping review
Source: Health Qual Life Outcomes. 2024 Feb 16;22:18. doi: 10.1186/s12955-024-02232-3 (PMC10870459; doi:10.1186/s12955-024-02232-3)
Supplement: Supplementary file 1 — Additional file 1. [file 12955_2024_2232_MOESM1_ESM.docx]

**Table Supplementary 1.**

| **Database** | **Descriptors** |
| --- | --- |
| PUBMED | (CHILDREN OR CHILD OR INFANT) AND (ADOLESCENTS OR TEENAGER) AND (“NEUROMUSCULAR DISEASE” OR “NEUROMUSCULAR DISORDER” OR NEUROMUSCULAR) AND (“QUALITY OF LIFE”)  **Additional filters:** publication period between 2012 and 2022; Languages: Portuguese, Spanish and English; Main topic; Full texts. |
| EMBASE | (CHILDREN OR CHILD OR INFANT) AND (ADOLESCENTS OR TEENAGER) AND (“NEUROMUSCULAR DISEASE” OR “NEUROMUSCULAR DISORDER” OR NEUROMUSCULAR) AND (“QUALITY OF LIFE”)  **Additional filters:** publication period between 2012 and 2022; Languages: Portuguese, Spanish and English; Main topic; Full texts. |
| SCOPUS | (CHILDREN OR CHILD OR INFANT) AND (ADOLESCENTS OR TEENAGER) AND (“NEUROMUSCULAR DISEASE” OR “NEUROMUSCULAR DISORDER” OR NEUROMUSCULAR) AND (“QUALITY OF LIFE”)  **Additional filters:** publication period between 2012 and 2022; Languages: Portuguese, Spanish and English; Main topic; Full texts. |
| LILACS | (CHILDREN OR CHILD OR INFANT) AND (ADOLESCENTS OR TEENAGER) AND (NEUROMUSCULAR DISEASE OR NEUROMUSCULAR DISORDER OR NEUROMUSCULAR) AND (QUALITY OF LIFE)  **Additional filters:** publication period between 2012 and 2022; Languages: Portuguese, Spanish and English; Main topic; Full texts. |
| SCIELO | (CHILDREN OR CHILD OR INFANT) AND (ADOLESCENTS OR TEENAGER) AND (“NEUROMUSCULAR DISEASE” OR “NEUROMUSCULAR DISORDER” OR NEUROMUSCULAR) AND (“QUALITY OF LIFE”)  **Additional filters:** publication period between 2012 and 2022; Languages: Portuguese, Spanish and English; Main topic; Full texts. |
| Google Scholar | (CHILDREN OR CHILD OR INFANT) AND (ADOLESCENTS OR TEENAGER) AND (“NEUROMUSCULAR DISEASE” OR “NEUROMUSCULAR DISORDER” OR NEUROMUSCULAR) AND (“QUALITY OF LIFE”);pdf  **Additional filters:** publication period between 2012 and 2022; |
